# Hemoglobin May Contribute to Sex Differences in Mortality among HIV-Infected Persons in Care

**DOI:** 10.1371/journal.pone.0044999

**Published:** 2012-09-13

**Authors:** Vlada V. Melekhin, Bryan E. Shepherd, Samuel E. Stinnette, Peter F. Rebeiro, Megan M. Turner, Timothy R. Sterling

**Affiliations:** 1 Division of Infectious Diseases, Department of Medicine, Vanderbilt University School of Medicine, Nashville, Tennessee, United States of America; 2 Department of Biostatistics, Vanderbilt University Medical Center, Nashville, Tennessee, United States of America; 3 Comprehensive Care Center, Nashville, Tennessee, United States of America; 4 Center for Health Services Research, Vanderbilt University School of Medicine, Nashville, Tennessee, United States of America; University of Hyderabad, India

## Abstract

**Background:**

Some retrospective studies have found that HIV-infected women have a higher mortality risk than men after adjusting for baseline characteristics, while others have not. Anemia is a known predictor of HIV-related mortality. We assessed whether anemia contributed to the sex difference in mortality in our cohort.

**Methods:**

We conducted a retrospective cohort study among HIV-infected persons in care at the Comprehensive Care Center (Nashville, TN) between 1998 and 2009. Cox proportional hazards models compared time from first clinic visit to death and AIDS-defining events (ADE), adjusted for baseline characteristics with and without baseline hemoglobin.

**Results:**

Of 3,633 persons, 879 (24%) were women. Women had lower median baseline hemoglobin compared to men: 12.4 g/dL (inter-quartile range (IQR) 11.3–13.4) vs. 14.4 (IQR 13.1–15.5), respectively (*P*<0.001). In multivariable models without hemoglobin, the risk of death was higher among women: hazard ratio (HR) 1.46 (95% confidence interval (CI) 1.17, 1.82; *P* = 0.001). In multivariable models with hemoglobin, the risk of death in women was diminished and no longer statistically significant: HR 1.2 (95% CI 0.93, 1.55; *P* = 0.17). The risk of ADE was higher among women in both models, but not statistically significant: HR 1.1 (95% CI 0.85–1.42; *P = *0.46) in the model without hemoglobin and 1.11 (95% CI 0.82–1.48; *P* = 0.50) in the model with hemoglobin. Hemoglobin was a strong predictor of death: HR 0.88 per 1 g/dL increase (95% CI 0.83, 0.93; *P*<0.001).

**Conclusion:**

In our study population of HIV-infected persons in care, women had lower baseline hemoglobin, and lower hemoglobin contributed to their higher risk of ADE and death.

## Introduction

In the highly active antiretroviral therapy (HAART) era, studies have shown inconsistent results regarding possible sex differences in mortality and HIV disease progression. Studies from the United States have found that women have an increased risk of HIV progression. [1], [2] Data from European cohorts have indicated a lower risk of disease progression among women. [3], [4] However, other studies have not found major sex differences in mortality or HIV disease progression. [5]–[7].

We found in an observational cohort study of persons in care from 1998 to 2005 that women had higher mortality rates than men at the same stage of HIV disease after accounting for receipt of HAART during study follow-up. [8] Similar results have been seen in other studies during the HAART era, [1], [9] although the sex differences in mortality were largely attributed to differences in HAART use [1] and clinical HIV disease stages [9] between sexes. None of these analyses adjusted for hemoglobin.

Anemia is a known predictor of HIV-related mortality in resource-limited and -replete settings, [10]–[18] and recovery from anemia is associated with improved survival. [15], [16] HIV-1-infected women are more likely to be anemic compared to men. [14], [19] Therefore, we sought to assess whether anemia contributed to the sex difference in mortality, as well as progression to AIDS-defining events, in our cohort of HIV-infected persons in care, with four additional years of data.

## Materials and Methods

### Study Cohort

This was a retrospective cohort study. The cohort was defined as HIV-1-infected persons in care (>1 visit) at the Comprehensive Care Center in Nashville, Tennessee, between 1 January 1998 and 31 December 2009. The cohort was an extension of the previously described cohort. [8].

**Table 1:** Demographic and clinical characteristics of the study population.

Characteristic	Study Population,N = 3,633	Women,N = 879	Men,N = 2,754	*P* a
Age at first clinic visit, median (IQR), years	38 (31–44)	37 (29–44)	39 (32–45)	0.001
Race/Ethnicity, no. (%)				<0.001
Caucasian	1,972 (54.3)	339 (38.6)	1633 (59.3)	
African-American	1,350 (37.2)	452 (51.4)	898 (32.6)	
Hispanic	168 (4.6)	37 (4.2)	131 (4.8)	
African	81 (2.2)	41 (4.7)	40 (1.5)	
Unidentified	21 (0.6)	4 (0.5)	17 (0.6)	
Other	31 (0.9)	7 (0.9)	24 (1.0)	
Baseline CD4+ lymphocyte count b, median (IQR), cells/mm^3^	325 (150–522)	360 (190–594)	316 (139.5–504)	<0.001
Missing, no. (%)	74 (2)	20 (2.3)	54 (2)	0.57
Baseline CD4+ lymphocyte percentage b, median (IQR), percent	21 (12–30)	24 (15–33)	20 (12–29)	<0.001
Missing, no. (%)	74 (2)	21 (2.4)	53 (1.9)	0.40
Baseline HIV-1 RNA b, median (IQR), log_10_ copies/mL	4.33 (3.07–4.94)	4.13 (3.03–4.78)	4.39 (3.07–4.99)	<0.001
Missing, no. (%)	69 (1.9)	15 (1.7)	44 (1.6)	0.82
HAART use at first clinic visit, no. (%)	1,189 (32.7)	243 (27.6)	946 (34.4)	<0.001
History of ADE, no. (%)	482 (13.3)	90 (10.2)	392 (14.2)	0.002
Prior non-HAART ART use, no. (%)	502 (13.8)	131 (15)	371 (13.5)	0.28
Prior HAART use, no. (%)	1,189 (32.7)	243 (27.7)	946 (34.4)	<0.001
Proportion of follow-up time while on HAART, median (IQR), %	68.7 (0–97.6)	59.7 (5.1–94.8)	71.3 (0–98)	0.02
Injection drug use c, no. (%)	369 (10.6)	104 (11.8)	265 (9.6)	0.06
History of HCV infection^d^, no. (%)	408 (11.2)	123 (14)	285 (10.4)	0.003
Year of enrollment in care, median (IQR), year	2004 (2001–2007)	2004 (2001–2007)	2004 (2001–2007)	0.46
Baseline hemoglobin, median (IQR), g/dLMissing, no. (%)	14 (12.5–15.2)492 (13.5)	12.4 (11.3–13.4)117 (13.3)	14.4 (13.1–15.5)375 (13.6)	<0.0010.18
Anemia, no. (%)	833 (23.0)	287 (32.7)	546 (19.8)	<0.001
Mild	567 (15.6)	208 (26.0)	359 (13)	
Moderate	226 (6.2)	53 (6.0)	173 (6.3)	
Severe	20 (0.6)	6 (0.7)	14 (0.5)	

Note: IQR: interquartile range. HAART: highly active antiretroviral therapy. HCV: hepatitis C virus infection. ADE: AIDS-defining event. ART: antiretroviral therapy.

a Continuous data were compared by Kruskal-Wallis test. Categorical data were compared by 2-sided Fisher’s exact test.

b Baseline values were defined as the most recent values <180 days prior to first clinic visit.

c History of injection drug use and other illicit drug use.

d Established HCV diagnosis.

Clinical data were entered into an electronic medical record by medical providers at the time of the patient encounter, automated data upload (e.g., laboratory results), or clinic personnel (e.g., deaths). Laboratory and antiretroviral therapy data (including medication start and stop dates) were validated by systematic chart review. All events were reviewed and confirmed by study investigators (PFR, DAR, SES, and MMT). An Endpoints Committee reviewed all questionable diagnoses. The informed consent process was waived in accordance with the Code of Federal Regulations (CFR) 45 CFR 46.116 (d). The study and the waiver of consent were approved by the Vanderbilt Institutional Review Board.

**Figure 1:**
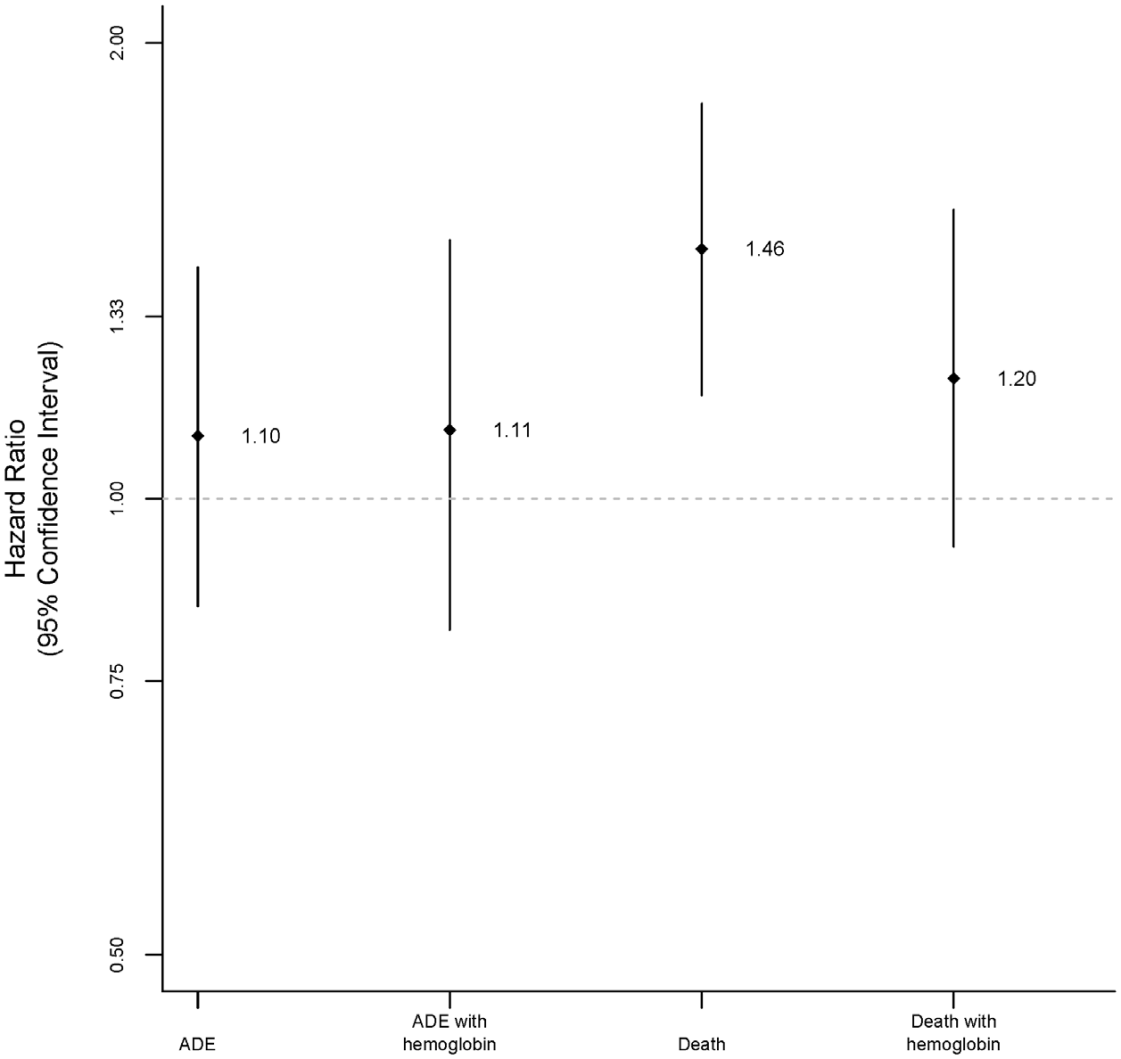
Hazard ratios for women compared to men for AIDS-defining events (ADE) and death.

**Table 2:** Cox proportional hazards model of progression to death.

Covariates	Univariate Analysis,HR* (95% CI)	*P*	Multivariable Analysis,without hemoglobin,HR (95% CI)	*P*	Multivariable Analysis,with hemoglobin,HR (95% CI)	*P*
Age at first clinic visit, per 1 year	1.04 (1.03–1.04)	<0.001	1.04 (1.03–1.05)	<0.001	1.04 (1.03–1.05)	<0.001
Baseline CD4+ lymphocyte count,per 1 cell/mm^3^	0.99 (0.99–0.99)	<0.001	0.99 (0.99–0.99)	0.01	0.99 (0.99–0.99)	0.01
Baseline CD4+ lymphocyte percentage, per 1 percent	0.96 (0.96–0.97)	<0.001	0.99 (0.98–1.01)	0.23	0.99 (0.98–1.01)	0.66
Baseline HIV RNA, per 1log_10_ copies/mL	1.38 (1.26–1.50)	<0.001	1.20 (1.06–1.35)	0.003	1.14 (1.01–1.30)	0.04
Baseline hemoglobin, per 1 g/dL	0.81 (0.77–0.85)	<0.001	NA	NA	0.88 (0.83–0.93)	<0.001
Race/Ethnicity	0.93 (0.84–1.00)	0.07	0.94 (0.86–1.03)	0.20	0.93 (0.84–1.03)	0.17
HAART use at first clinic visit	0.91 (0.74–1.12)	0.36	0.87 (0.64–1.18)	0.38	0.81 (0.58–1.13)	0.22
History of HCV	1.73 (1.37–2.17)	<0.001	1.31 (1.00–1.70)	0.05	1.32 (0.99–1.75)	0.66
Injection drug use	2.18 (1.75–2.72)	<0.001	1.59 (1.23–2.06)	<0.001	1.60 (1.21–2.11)	0.001
Prior HAART use	1.05 (0.87–1.27)	0.63	1.34 (1.03–1.75)	0.03	1.48 (1.12–1.97)	0.01
Prior non-HAART ART use	1.56 (1.26–1.94)	<0.001	1.24 (0.98–1.57)	0.07	1.34 (1.04–1.74)	0.02
History of ADE	2.24 (1.82–2.76)	<0.001	1.44 (1.14–1.83)	0.002	1.21 (0.93–1.57)	0.15
Sex, men as a reference group	1.07 (0.87–1.31)	0.54	1.46 (1.17–1.82)	0.001	1.20 (0.93–1.55)	0.17
Year of cohort entry	0.85 (0.82–0.88)	<0.001	0.86 (0.82–0.89)	<0.001	0.87 (0.83–0.90)	<0.001

* HR: Hazard ratio.

**Table 3:** Cox proportional hazards models of progression to ADE.

Covariates	Univariate Analysis,HR (95% CI)	*P*	Multivariable Analysis,without hemoglobin, HR (95% CI)	*P*	Multivariable Analysis,with hemoglobin,HR (95% CI)	*P*
Age at first clinic visit, per 1 year	1.02 (1.00–1.03)	0.003	1.02 (1.01–1.03)	0.02	1.02 (1.01–1.03)	0.02
Baseline CD4+ lymphocyte count,per 1 cell/mm^3^	0.99 (0.99–0.99)	<0.001	0.99 (0.99–0.99)	0.002	0.99 (0.99–0.99)	0.02
Baseline CD4+ lymphocyte percentage, per 1 percent	0.96 (0.95–0.97)	<0.001	0.99 (0.98–1.01)	0.74	0.99 (0.97–1.01)	0.37
Baseline HIV RNA, per 1log_10_ copies/mL	1.33 (1.18–1.50)	<0.001	1.18 (1.01–1.38)	0.04	1.12 (0.94–1.34)	0.22
Baseline hemoglobin, per 1 g/dL	0.89 (0.84–0.93)	<0.001	NA	NA	0.95 (0.90–1.02)	0.14
Race/Ethnicity	1.01 (0.92–1.11)	0.86	0.95 (0.86–1.06)	0.37	0.94 (0.84–1.05)	0.27
HAART use at first clinic visit	0.96 (0.74–1.25)	0.79	1.19 (0.84–1.69)	0.32	1.03 (0.71–1.52)	0.86
History of HCV	0.74 (0.55–1.00)	0.05	0.87 (0.62–1.22)	0.42	0.92 (0.65–1.30)	0.64
Injection drug use	0.83 (0.62–1.11)	0.22	0.9 (0.65–1.25)	0.54	0.88 (0.63–1.24)	0.48
Prior HAART use	0.92 (0.73–1.16)	0.48	0.85 (0.63–1.14)	0.28	0.87 (0.63–1.2)	0.41
Prior non-HAART ART use	0.82 (0.63–1.07)	0.15	1.26 (0.93–1.7)	0.13	1.55 (1.11–2.17)	0.01
History of ADE	1.50 (1.19–1.89)	0.001	0.91 (0.71–1.18)	0.5	0.85 (0.64–1.12)	0.25
Sex, men as a reference group	0.91 (0.72–1.15)	0.43	1.1 (0.85–1.42)	0.46	1.11 (0.82–1.48)	0.5
Year of cohort entry	1.20 (1.15–1.24)	<0.001	1.19 (1.14–1.24)	<0.001	1.2 (1.14–1.25)	<0.001

### Definition of Study Exposure

Data collected included patient characteristics such as age, sex, race, baseline CD4+ lymphocyte count and percentage (the earliest tests available at first clinic visit±90 days), baseline HIV-1 RNA, hepatitis C virus (HCV) established diagnosis as determined by clinic providers based on HCV-positive antibody and/or HCV viral load, history of injection drug use (IDU), history of AIDS-defining event (ADE), prior use of HAART and non-HAART ART, year of enrollment in care, and baseline hemoglobin.

HAART was defined as regimens of ≥7 days duration that contained at least three antiretroviral medications. Non-HAART antiretroviral therapy (ART) included mono- or dual-nucleoside reverse transcriptase inhibitor (NRTI) therapy.

For Table 1, anemia was defined as severe (hemoglobin <8 g/dL), moderate (≥8-<11 g/dL for men and ≥8-<10 g/dL for women), and mild (≥11-<13 g/dL for men and ≥10-<12 g/dL for women).

Loss to follow-up was defined as having no provider visit within 1 year prior to the end of the study period or date of death, whichever occurred first.

### Definition of Study Outcomes

Outcomes of interest were time to death and ADE at any time during follow-up. ADEs were based on the 1993 US Centers for Disease Control and Prevention (CDC) classification criteria, [20] excluding CD4+ lymphocytes <200 cells/mm^3^. Information on death was obtained from medical records. The records were routinely updated based on reports from families, local newspapers, hospitals, and the Social Security Death Index.

### Follow-up

Follow-up started at first clinic visit. For the time to death analyses, follow-up ended at the date of death, last clinic encounter, or 31 December 2009. For the time to ADE analyses, follow-up ended with the first ADE or death, last clinic encounter, or 31 December 2009.

### Statistical Analyses

STATA IC (version 10.1; Stata Corporation) was used for all analyses. Continuous variables were compared with rank sum tests, and categorical variables were compared with Fisher’s exact test.

Cox proportional hazards models compared rates of death (due to all causes) and ADE according to sex, both unadjusted and adjusted for baseline variables (see below), and with and without baseline hemoglobin. Variables were chosen for inclusion in the model *a priori* based on clinical relevance and included age at first clinic visit, sex, race, baseline CD4+ lymphocyte count and percentage, baseline HIV-1 RNA, HAART use at first clinic visit, HAART and non-HAART ART use prior to first clinic visit, ADE prior to first clinic visit, history of IDU or HCV co-infection, and year of enrollment. Primary analyses were conducted using only those patients with non-missing data for all covariates included in the model. Secondary analyses included the above multivariable analyses while including an interaction term between sex and hemoglobin and multiple imputation techniques to address missing covariate data. Multiple imputation was performed using the default settings of the ice and mim functions in STATA with 10 imputation replications.

## Results

### Patient Characteristics

Of 3,633 persons in care at the Comprehensive Care Center between 1 January 1998 and 31 December 2009, 879 (24.2%) were women. Table 1 shows the demographic and clinical characteristics of the study subjects. Compared to men, women were younger and more likely to be African-American, had higher baseline CD4+ lymphocyte count and CD4+ lymphocyte percentage, lower baseline HIV-1 RNA, lower proportion with prior ADE and prior HAART exposure, and lower proportion of follow-up time on HAART while in care. Of those with available data (82.6%), women had lower baseline hemoglobin and were more likely to have mild anemia. There were no statistically significant differences by sex in the proportion of persons on HAART at the first clinic visit, proportion with history of IDU and HCV, prior non-HAART ART exposure, and year of enrollment. Median (inter-quartile range (IQR)) follow-up was similar by sex: 37.2 (12.8–75.2) months in women and 35.1 (11.9–73.1) months in men (*P* = 0.43). A higher proportion of men 914 (33.2%) than women 239 (27.2%) were lost to follow-up (*P* = 0.001).

### ADE and Death Analyses

Despite differences in baseline characteristics, the proportions of men and women who developed an ADE (276 (10%) men and 97 (11%) women) or died (353 (13%) men and 123 (14%) women) were similar. Among persons who had an ADE, there were no statistically significant differences in the proportion of those with missing hemoglobin (15% among men and 19% among women). There were also no sex differences in the proportion with missing hemoglobin among those who died (19% among men and 17% among women).

In unadjusted time-to-event analyses, event rates were also similar between women and men: hazard ratio (HR) for women 1.07 (95% confidence interval (CI) 0.87, 1.31; *P* = 0.54) for death and 0.91 (95% CI 0.72, 1.15; *P = *0.43) for ADE (Figure 1, Table 2 and 3).

In multivariable analyses that included important baseline characteristics (see *Methods*) excluding baseline hemoglobin, the risks of death (HR 1.46, 95% CI 1.17, 1.82; *P*<0.001) were higher among women than men. When the above analyses were repeated including baseline hemoglobin, the risk of death (HR 1.20, 95% CI 0.93, 1.55; *P* = 0.17) among women was not statistically different compared to men.

The risk of ADE was also higher, though not statistically different, among women compared to men in both models: HR 1.10 (95% CI 0.85, 1.42; *P* = 0.46) in the model without baseline hemoglobin and HR 1.11 (95% CI 0.82, 1.48; *P* = 0.5) in the model with baseline hemoglobin (Table 2 and 3).

Increased hemoglobin was significantly associated with a lower risk of death, but not ADE in the multivariable models: HR 0.88 per 1 g/dL increase (95% CI 0.83, 0.93; *P*<0.001) and 0.95 per 1g/dL increase (95% CI 0.90, 1.0; *P* = 0.14), respectively (Table 2 and 3). There was little evidence of an interaction between hemoglobin and sex on death or ADE: *P* = 0.46, and 0.28, respectively.

The results were similar in all of the above analyses when we limited the analyses to those without missing hemoglobin (for the model without hemoglobin, the HR of death for women was 1.53 (95% CI 1.18, 1.98; *P* = 0.001)) and multiply imputed missing baseline laboratory values (for the model without hemoglobin, the HR of death for women was 1.42 (95% CI 1.12, 1.81; *P* = 0.004); for the model with hemoglobin, the HR of death for women was 1.27 (95% CI 0.98, 1.64; *P* = 0.07)).

Results were also similar when we included the proportion of follow-up time on HAART: for the model without hemoglobin, the HR of death was 1.42 (95% CI 1.14, 1.77; *P = *0.002); for the model with hemoglobin, the HR of death was 1.16 (95% CI 0.90, 1.50; *P = *0.26).

## Discussion

In this retrospective cohort study of HIV-infected persons in care between 1998 and 2009, women had higher rates of death than men at the same stage of HIV disease when not accounting for baseline hemoglobin. However, when baseline hemoglobin was considered, the association between sex and risk of death was diminished. Low hemoglobin therefore accounted for some, though not all, of the increased risk of death in women.

Similar to our earlier study [8], women presented to care at an earlier HIV disease stage. This may explain why women had lower proportion of follow-up time on HAART, since they may have been less likely to meet indications for HAART. It is possible that women had less access to HAART when they met the criteria for treatment initiation, or higher rates of HAART discontinuation, [21] and thus a higher risk of death. However, less time on HAART does not seem to be explaining the increased risk of death in our cohort for women.

Moreover, women had higher rates of anemia compared to men. This finding has been seen in other studies [14], [19]. This in turn could explain higher rates of mortality among women in the analyses that did not include hemoglobin. [10], [18] In the current analyses, baseline hemoglobin contributed to the sex differences in mortality. These findings differ from another study in which there were no statistically significant sex differences in disease progression despite lower baseline hemoglobin in women. [14].

Unlike the previous study from our center, this study assessed the association between sex and HIV disease progression and mortality after controlling for baseline hemoglobin. Although hemoglobin has long been known to be a predictor of HIV disease progression, there have been conflicting results regarding the effect of sex on disease progression after adjusting for hemoglobin [13], [16], [17], [22].

Our study had some limitations. First, our study may lack the necessary sample size to statistically detect a true difference in the risk of HIV disease progression between men and women in multivariable survival analyses that account for hemoglobin and other covariates. Larger studies are warranted. Second, residual confounding by indication for HIV treatment and unmeasured confounding might remain despite statistical methods to account for the baseline differences between men and women. Third, the current study cannot prove causality of the association between hemoglobin and HIV disease progression. Lower hemoglobin might be a marker of generalized immune activation [23] that in turn independently leads to HIV disease progression [24]. Fourth, we used a single baseline hemoglobin value that ignored changes in hemoglobin over the course of follow-up.

In summary, comparing men and women at a similar stage of disease at presentation to care, women had higher mortality risk than men. However, the sex difference in survival could be explained in part by the sex difference in baseline hemoglobin. Further studies are needed to assess these differences in mortality in larger, more representative cohorts before these findings can be extrapolated to other settings. Given different rates of anemia between men and women, future statistical analyses of mortality should include hemoglobin. In clinical practice, we may need to pay especially close attention to managing anemia among women as it may contribute to the overall increased mortality among women.
